# *Kelussia odoratissima *potentiates cytotoxic effects of radiation in HeLa cancer cell line

**Published:** 2017

**Authors:** Azar Hosseini, Shima Saeidi Javadi, Azar Fani-Pakdel, Seyed Hadi Mousavi

**Affiliations:** 1 *Pharmacological Research Center of Medicinal Plants, MashhadUniversity of Medical Sciences, Mashhad, Iran *; 2 *Department of Pharmacology, School of Medicine, Mashhad University of Medical Sciences, Mashhad, Iran*; 3 *Department of Oncology, Omid Hospitsal, Mashhad, Iran*

**Keywords:** HeLa cell line, Kelussia odoratissima, Cytotoxicity, Radiotherapy, Apoptosis

## Abstract

**Objective:**

Cervical cancer is the second most common cause of death from cancer in women throughout the world. The aim of this study was to evaluate the cytotoxic activity of *Kelussia odoratissima* (*K. odoratissima*) extract associated with radiotherapy in cervical cancer cells (HeLa cell line).

**Materials and Methods:**

Different concentration of the extract (25-500µg/ml) was tested in HeLa cell lines. Cell cytotoxicity of the extract and the effects of the extract on radiation (2Gy/min)-induced damages were assessed by MTT assay. Apoptosis was assessed using flow cytometric analysis.

**Result:**

*K. odoratissima* decreased cell viability in HeLa cell line in a concentration and time-dependent manner. When compared to the control, *K. odoratissima* induced a sub-G1 peak in the flow cytometry histogram of treated cells, indicating that apoptotic cell death is involved in *K. odoratissima*-induced toxicity. It was also shown that *K. odoratissima* sensitizes cells to radiation-induced toxicity.

**Conclusion:**

Our result showed the extract increased the radiation effect. This observation may be related to the presence of active compounds such as phthalides and ferulic acid.

## Introduction

Cervical cancer is common reason of death in women worldwide (Bosch et al., 2002 ▶). The important treatment for cervical cancer, is radiotherapy especially for locally advanced tumors, and led to cell death in tumorous tissues (Dunne-Daly CF, 1999 ▶). In the later stages of cancer, radiotherapy fails because of the presence of the radio-resistant tumor cells. The cytotoxic agent is very important in radiobiology because of increasing the oxidative damage of the tumor cells (Olive, 1998 ▶). Radiosensitizers are used to increase tumor cell killing because of they have less effect on normal tissues. Most of the radiosensitizers are chemical compounds which exhibit toxicity (Wardman, 2007 ▶). Natural products are active compounds used for treatment of diseases since ancient times. The studies have shown that dietary phytochemicals has shown useful effects in a treatment of pathological situation when used alone or in combination with radiation (Bhoslea et al., 2005 ▶). Phytochemicals can act as radiosensitizers in a variety of cancer cell lines, *in vitro* and *in vivo* (Bhoslea et al., 2005 ▶). Phytochemicals amplify the effects of irradiation by several modes including toxic reactions of free radicals, overriding cell cycle arrest, and inducing apoptosis (Zoberi et al., 2002 ▶). Umbelliferae has more than 450 genera and near 3700 species in the world (She et al., 2005 ▶). Kelussia is identified genus of this family and is represented by only one species, *Kelussia odoratissima *Mozaff. is found in Iran (Mozaffarian 2003 ▶). This medicinal plant is endemic to a limited area in western of Iran and is popularly called Karafse-Koohi. In traditional medicine, aerial parts of the plant are used for sedative. Also, *K. odoratissima* is consumed to treat hypertension, cardiovascular diseases and inflammation ulcers (Ahmadi et al., 2007 ▶). The antioxidant effects of the methanolic extract of the plant were investigated by several methods (Ahmadi et al., 2007 ▶). In the present study, for the first time, we analyzed the sensitizing effects of *K. odoratissima *when co-administered with γ–radiation, in human cervical cancer cell line.

## Materials and Methods

**Cell line and agents**

Human cervical cancer cell line (HeLa) was bought from Pasteur Institute (Tehran, Iran). Propidium iodides (PI), sodium citrate, Triton X-100, 4, 5-Dimethylthiazol-2-yl, 2, 5-diphenyl tetrazolium (MTT) and Dulbecco’s Phosphate-buffered saline (PBS) were prepared from Sigma (St Louis, MO, USA). Fetal bovine serum (FBS), Glucose-high Dulbecco’s modified Eagle’s medium (DMEM), penicillin and streptomycin were purchased from Gibco (Grand Island, NY). Dimethyl sulfoxide (DMSO) was purchased from Merck. 

**Preparation of Extract**

Aerial parts of *K. odoratissima* were collected from Zard-Kooh Mountains, Charmahal-e-Bakhtiari province, Iran and identified by the herbarium of Ferdowsi University of Mashhad, Mashhad, Iran (voucher specimen number: 35205). The plant was dried, powdered and subjected to extraction with 70% ethanol in a Soxhlet apparatus for 48 hr. The extract was then dried on a water bath, dissolved in DMSO and kept in freezer at -20^o^C until use.

**Cell culture**

HeLa cells were cultured in high glucose DMEM (4.5 g/l) supplemented with 10% FBS and *100 units*/*ml penicillin* and *100* micrograms/*ml streptomycin*. All cells were maintained in a humidified atmosphere (90%) containing 5% CO_2_ at 37^0^C.

**Cell proliferation (MTT) Assay**

Cells (5000/well) were seeded in 96-well culture plates and after 24 hr, the cells were treated with different concentrations of the extract (25-500µg/ml) and then, incubated for another 24, 48 and 72 hr. Cell viability was assessed by MTT assay 24, 48 and 72 hr after treatment with the extract. MTT solution in phosphate-buffered saline (5 mg/ml) was added to each well at a final concentration of 0.05%. After 3 hr, the formazan precipitate was dissolved in DMSO. The absorbance at 570 and 620 nm (background) was measured using a StatFAX303 plate reader. All treatments were carried out in triplicate.

**Combined plant extracts and irradiation cytotoxicity assay**

In the second experiment, cells were plated in 96-well plates at a 5×10^3^ cells/well density and treated with the extracts as described above. After a 24 hr-treatment, cells were washed and maintained in PBS for irradiation. The irradiation was performed with a 60Co unit at a dose of 2 Gy, γ-rays during exponential cell growth as monolayers in 96-microwell plate (Magné et al., 2002 ▶). As controls, cells were treated only with radiation, and one plate was seeded but left without irradiation. Immediately after the irradiation, PBS was removed and cells were maintained in culture medium without extract. Cell viability was assessed by MTT assay, 66 hr after the irradiation (Torres et al., 2011 ▶).

**Cell apoptosis assay**

Apoptotic cells were detected using PI staining of small DNA fragments followed by flow cytometry. It has been reported that a sub-G1 peak that is reflective of DNA fragmentation can be observed following the incubation of cells in a hypotonic phosphate-citrate buffer containing a quantitative DNA-binding dye, such as PI. Apoptotic cells that have lost DNA will take up less stain and appear on the left side of the G1 peak in the histogram. Briefly, HeLa cells were seeded in wells of a 24-well plate, overnight. Then, cells were treated with different concentrations of the extract. Floating and adherent cells were then harvested and incubated with 750 µl of a hypotonic buffer (50 µg/ml PI in 0.1% sodium citrate with 0.1% Triton X-100) at 4 °C, in the dark, overnight. Next, flow cytometry was carried out using a FACScan flow cytometer (Becton Dickinson). A total of 10000 events were acquired with FACS.

**Statistical analysis**

One-way analysis of variance (ANOVA) followed by Bonferroni’s *post-hoc* test for multiple comparisons, was used for data analysis. All results were expressed as mean *±* SEM. A p*<*0*.*05 was considered statistically significant.

## Results

**Cytotoxic effect of** ***K. odoratissima*** **on cell viability**

**Figure 1.**
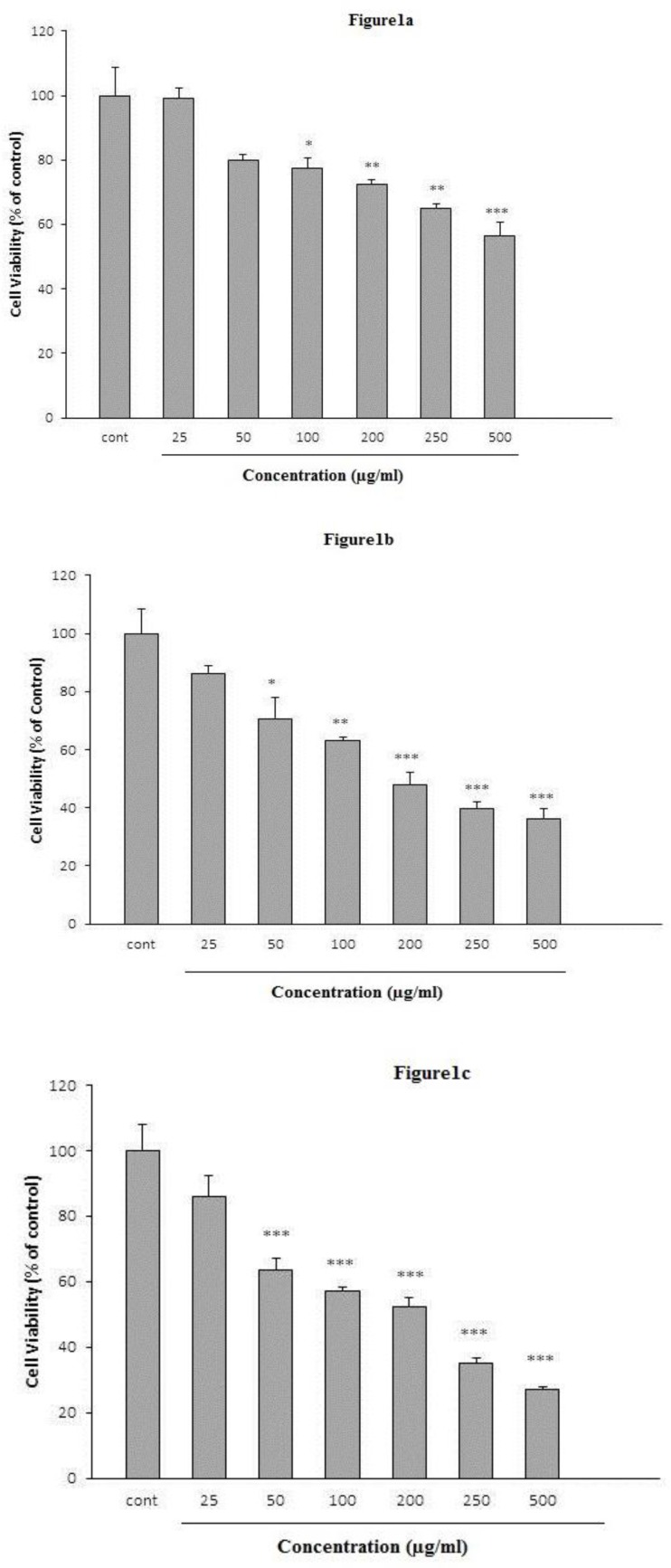
The cytotoxic effect of *K. odoratissima* on HeLa cells. HeLa cells were treated with various concentrations of *K. odoratissima* for 24 (Figure 1a), 48 (Figure 1b), and 72 hr (Figure 1c). Viability was quantitated by the MTT assay. The data are expressed as mean±SEM (n = 3). *p<0.05, **p<0.01, and *** p< 0.001

 For evaluation of the toxic effects of *K. odoratissima*, HeLa cells were incubated with different concentrations of the extract (25-500 µg/ml), and cell viability was determined 24, 48 and 72 hr after treatment. The extract decreased HeLa cells viability as a dose and time-dependently. Cell viability reduced after 24 hr at the doses of 100 (77.27±3.24%, p<0.05), 200 (72.39±1.5%, p<0.01), 250 (65.14±1.34%, p<0.01) and 500 µg/ml (56.4±4.46%, p<0.001) (Figure 1a). 

After 48 hr, cell viability decreased at the doses of 50 (70.39±7.74%, p<0.05), 100 (62.97±1.48%, p<0.01), 200 (47.95±4.09%, p<0.001), 250 (39.67±2.17%, p<0.001) and 500 µg/ml (36.19±3.46%, p<0.001) (Figure 1b). Cell viability reduced after 72 hr at the doses of 50 (63.68±3.5%, p<0.001), 100 (57.21±1.33%, p<0.001), 200 (52.22±2.82%, p<0.001), 250 (35±1.7%, p<0.001) and 500 µg/ml (27±1.08%, p<0.001) (Figure 1c). 

**Cytotoxic effect of co-administration of irradiation and the extract in HeLa cell line**

**Figure 2:**
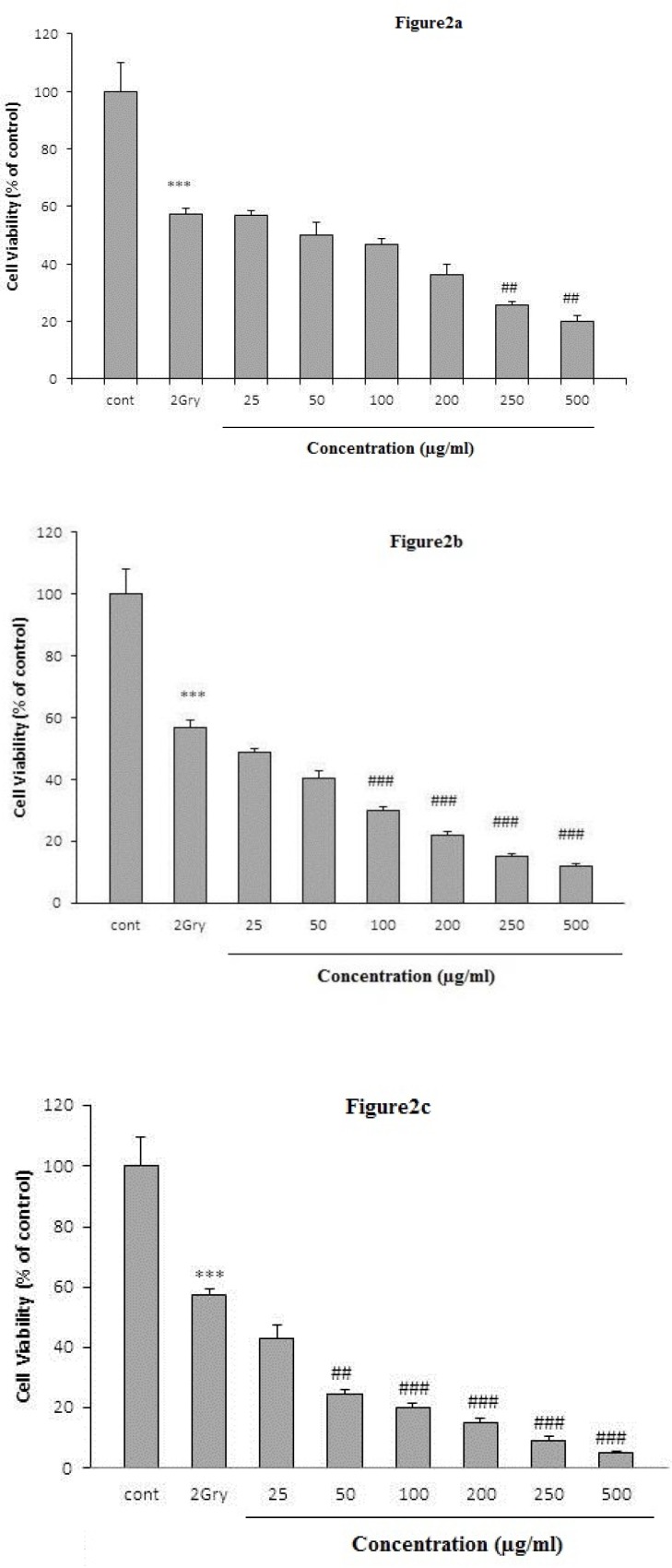
Evaluation of the cytotoxic effect of co-administration of irradiation and extract in HeLa cell line. HeLa cells were treated with different concentrations of *K. odoratissima* for 24 (Figure 2a), 48 (Figure 2b), and 72 hr (Figure e3c). Viability was quantitated by the MTT assay after 66 hr. The data are expressed as mean±SEM (n = 3). *** p<0.001 compared to control. ^##^ p<0.01 and ^###^ p< 0.001 compared to radiation group

 A single irradiation dose of 2 Gy/min reduced cell viability in HeLa cell line (57±2%, p<0.001). To investigate whether the extract can be an adjuvant treatment to radiotherapy, we treated the cells with plant extract 24, 48 and 72 hr before irradiation treatment. Co-administration of the irradiation and the extract improved the cytotoxic response in the cell line (Figure 2a, 2b and 2c). After a 24 hr-treatment, extract potentiated the effect of radiation at doses of 250 (25.62±1.44%, p<0.001) and 500 µg/ml (20±2.1%, p<0.001) (Figure 2a). After 48 (Figure 2b) and 72 h (Figure 2c), the extract increased the radiation effect at doses of 100-500 µg/ml and 50-500 µg/ml, respectively.

**Apoptosis-inducing activity of** ***K. odoratissima*** **in HeLa cell line**

**Figure 3:**
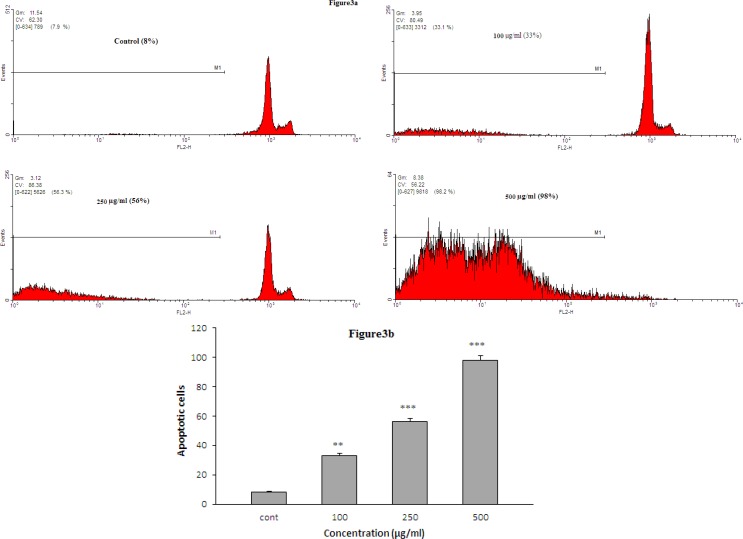
a) The role of apoptosis in *K. odoratissima* induced toxicity in HeLa cells. HeLa cells were treated with the extract for 24 hr. A sub-G1 peak, as an indicator of the presence of apoptotic cells, was induced in the *K. odoratissima* -treated cells but not in the control cells. b) The effects of the *K. odoratissima* on apoptosis in HeLa cells using PI staining and flow cytometry. **p<0.001 and ***p<0.001 versus control

To evaluate the apoptotic activity of the extract, HeLa cells were incubated with different concentrations (100, 250 and 500 µg/ml) of the extract. Cell apoptosis was determined 24 hr after treatment. As shown in Figures 3a and 3b, the extract increased cell apoptosis in HeLa cell line in a concentration-dependent manner after 24 hr. 

## Discussion

Cancer is a problem in developing and developed countries. The World Health Organization (February, 2014) has been reported that 8.2 million patients died from cancer in 2012. It has been also estimated that the number of annual cancer cases would increase from 14 million in 2012 to 22 million within the next two decades (WHO, 2014). Currently, the main treatments for cancer are chemotherapy, radiotherapy and surgery. Some of the mostly used chemotherapy drugs include methotrexate as an anti-metabolite, cisplatin and doxorubicin which interact with DNA, taxanes which are antitubulin agents, hormones and drugs that affect on molecular targests (Nussbaumer et al., 2011 ▶). But, using of these drugs in clinic led to several adverse effects such as hair loss, suppression of bone marrow, drug resistance, gastrointestinal lesions, neurologic dysfunction and cardiac toxicity (Nussbaumer et al., 2011 ▶; Monsuez et al., 2010 ▶; Dropcho 2011 ▶). As result, the researchers attempt to find new anticancer agents which have lower side effects and higher efficacy. The recent investigations have been shown natural products are appropriate sources for treatment of different diseases. Vinca alkaloids such as vinblastine and vincristine that are used in clinical as anti-cancer agents are derived from plant. Other anti-cancer drugs which are obtained from herbs are including paclitaxel, the epipodophyllotoxin derivative etoposide, and the camptothecin derivatives, topotecan and irinotecan (Cragg and Newman, 2005 ▶). These drugs act via different mechanisms for example Vinca alkaloids and paclitaxel inhibit microtubule assembly, supression of DNA topoisomerase II (etoposide) and DNA topoisomerase I inhibition such as campothecin derivatives. Also, the production of reactive oxygen species (ROS) may play role in anti-cancer effects of these drugs (Alexandre et al., 2006 ▶; Gorman et al., 1997 ▶; Alexandre et al., 2007 ▶). The induction of oxidative stress by pro-oxidant agents is a strategy for killing of cancer cells (Pelicano et al., 2004 ▶; Renschler 2004 ▶; Schumacker 2006 ▶; Lopez-Lazaro 2007 ▶). Carcinogenesis agents increase ROS level in cancer cells. Pro-oxidant agents increase the cellular levels of ROS, therefore, they can induce carcinogenic effects. Increasing of ROS level by pro-oxidant agents in cancer cells led to death of these cells. The most of natural products have anti-oxidant and pro-oxidant properties. For example, curcumin can act as cancer chemo-preventive, carcinogenic, and chemotherapeutic agents which depends on the applied concentration (Lopez-Lazaro 2007 ▶; Lopez-Lazaro 2008 ▶). The recent investigations have been reported that ionizing radiation increase the production of ROS in cells (Dal-Pizzol et al., 2003 ▶). Therefore, using of ionizing radiation may sensitize cancer cells to cytotoxic agents via oxidative stress increment. In this study, the cytotoxic effect of *K. odoratissima* in combination with radiation was evaluated for the first time. Our findings showed that the extract potentiated the effects of radiation. *K. odoratissima* belongs to umbelliferae. Phytochemical studies have shown that *K. odoratissima* contains coumarins, phenolics, flavonoids, terpenoids, phthalides and ferulic acid (Dewick 2011 ▶; Sajjadi et al., 2012 ▶). Recent studies have shown that some compounds such as phthalides and ferulic acid, potentiated the radiation effect (Qi et al., 2015 ▶; Bandugula and Prasad, 2013 ▶). Qi and co-workers showed combination of phthalides with radiation increased radio-sensitivity in human liver cancer cells via modulating caspase-dependent apoptosis protein (Qi et al., 2015 ▶). Also, ferulic acid, a dietary phenolic acid, increased radiosensitizing in NCI-H460 cells through a pro-oxidant mechanism (Reddy and Prasad, 2011 ▶). Combining glycolytic inhibition with plant-derived phenolics is a new approach being considered to selectively kill cancer cells. Plant phenolic compounds are as antioxidant agents, but they also have been shown pro-oxidant properties in cancer cells; this effect is related to acidic environment in cancer cells and the presence of high levels of peroxidases which act on phenolics and produce phenoxy radicals (Lee and Lee, 2006 ▶). Therefore, phenolic phytochemicals may play role in cancer therapy via increment of radio-sensitization in cancer cells (Garg et al., 2005 ▶). The levels of ROS in tumor cells is higher than normal cells, therefore they are more sensitive to oxidative stress generated by anti-cancer agents (Trachootham et al., 2009 ▶). Also programmed cell death increase in cancer cells by ROS. For example phenolic phytochemicals induce apoptosis in cancer cells via ROS generation (Garg et al., 2005 ▶). According to our results, the extract increased the radiation effect. This observation may be related to the presence of active compounds such as phthalides and ferulic acid. 

## References

[B1] Ahmadi F, Kadima M, Shahedi M (2007). Antioxidant activity of Kelussia odoratissima Mozaff in model and food systems. Food chem.

[B2] Alexandre J, Batteux F, Nicco C (2006). Accumulation of hydrogen peroxide is an early and crucial step for paclitaxel-induced cancer cell death both in vitro and in vivo. Int J Cancer.

[B3] Alexandre J, Hu Y, Lu W, Pelicano H, Huang P (2007). Novel action of paclitaxel against cancer cells: bystander effect mediated by reactive oxygen species. Cancer Res.

[B4] Bandugula VR, Prasad R (2013). 2-Deoxy-D-glucose and ferulic acid modulates radiation response signaling in non-small cell lung cancer cells. Tumor Biol.

[B5] Bhoslea SM, Huilgola NG, Mishra KP (2005). Enhancement of radiation-induced oxidative stress and cytotoxicity in tumor cells by ellagic acid. Clin Chim Acta.

[B6] Bosch FX, Lorincz A, Munoz N (2002). The casual relation between human papilloma virus and cervical cancer. J Clin Pathol.

[B7] Cragg GM, Newman DJ (2005). Plants as a source of anti-cancer agents. J Ethnopharmacol.

[B8] Dal-Pizzol F, Ritter C, Klamt F, Andrades M, da Frota ML Jr, Diel C (2003). Modulation of oxidative stress in response to γ-radiation in human glioma cell lines. J Neurooncol.

[B9] Dewick PM (2011). Medicinal Natural Products: A Biosynthetic Approach . West Sussex.

[B10] Dropcho EJ (2011). The neurologic side effects of chemotherapeutic agents. Continuum (Minneap Minn) 17.

[B11] Dunne-Daly CF (1999). Principles of radiotherapy and radiobiology. Semin Oncol Nurs.

[B12] Garg AK, Buchholz TA, Aggarwal BB (2005). Chemosensitization and radiosensitization of tumors by plant polyphenols. Antioxid Redox Signal.

[B13] Gorman A, McGowan A, Cotter TG (1997). Role of peroxide and superoxide anion during tumour cell apoptosis. FEBS Lett.

[B14] Lee KW, Lee HJ (2006). The roles of polyphenols in cancer chemoprevention. Biofactors.

[B15] Lopez-Lazaro M (2008). Anticancer and carcinogenic properties of curcumin: considerations for its clinical development as a cancer chemopreventive and chemotherapeutic agent. Mol Nutr Food Res.

[B16] Lopez-Lazaro M (2007). Dual role of hydrogen peroxide in cancer: Possible relevance to cancer chemoprevention and therapy. Cancer Lett.

[B17] Magné N, Fischel JL, Dubreuil A, Formento P, Marcié S, Lagrange JL, Milano G (2002). Sequence-dependent effects of ZD1839 (‘Iressa’) in combination with cytotoxic treatment in human head and neck cancer. Br J Cancer.

[B18] Monsuez J, Charniot JC, Vignat N, Artigou JY (2010). Cardiac side-effects of cancer chemotherapy. Inter J Cardiol.

[B19] Mozaffarian V (2003). Two new genera of Iranian Umbellifera. Bot Zhurn.

[B20] Nussbaumer S, Bonnabry P, Veuthey JL, Sandrine F (2011). Analysis of anticancer drugs: A review. Talanta.

[B21] Olive PL (1998). The role of DNA single and double strand breaks in cell killing by ionizing radiation. Radiat Res.

[B22] Pelicano H, Carney D, Huang P (2004). ROS stress in cancer cells and therapeutic implications. Drug Resist Updat.

[B23] Qi F, Zhao L, Zhou A, Zhang B, Li A, Wang Z, Han J (2015). The advantages of using traditional Chinese medicine as an adjunctive therapy in the whole course of cancer treatment instead of only terminal stage of cancer. BioScience Trends.

[B24] Reddy BV, Prasad NR (2011). 2-deoxy-D-glucose combined with ferulic acid enhances radiation response in non-small cell lung carcinoma cells. Cent Eur J Biol.

[B25] Renschler MF (2004). The emerging role of reactive oxygen species in cancer therapy. Eur J Cancer.

[B26] Sajjadi SE, Shokoohinia Y, Moayedi NS (2012). Isolation and Identification of Ferulic Acid From Aerial Parts of Kelussia odoratissima Mozaff. Jundishapur J Nat Pharm Prod.

[B27] Schumacker PT (2006). Reactive oxygen species in cancer cells: live by the sword, die by the sword. Cancer Cell.

[B28] She M, Pu F, Pan Z (2005). "Apiaceae". Flora of China.

[B29] Torres MA, Raju U, Molkentine D, Riesterer O, Milas L, Ang KK (2011). AC480, formerly BMS-599626, a pan Her inhibitor, enhances radiosensitivity and radioresponse of head and neck squamous cell carcinoma cells in vitro and in vivo. Invest. New Drugs.

[B30] Trachootham D, Alexandre J, Huang P (2009). Targeting cancer cells by ROS-mediated mechanisms: a radical therapeutic approach?. Nat Rev Drug Discov.

[B31] Wardman P (2007). Chemical Radiosensitizers for use in radiotherapy. Clin Oncol.

[B32] Zoberi I, Bradbury CM, Curry HA (2002). Radiosensitizing and antiproliferative effects of resveratrol in two human cervical tumor cell lines. Cancer Lett.

